# Circulating soluble receptor for advanced glycation end products and other factors in type 2 diabetes patients with colorectal cancer

**DOI:** 10.1186/s12902-020-00647-9

**Published:** 2020-11-13

**Authors:** Xiaohai Zhou, Ning Lin, Mingjie Zhang, Xiaoling Wang, Ye An, Qing Su, Peng Du, Bo Li, Hanbei Chen

**Affiliations:** 1 Department of Endocrinology, Xinhua Hospital affiliated with Shanghai Jiaotong University School of Medicine, 1665 Kongjiang Road, Yangpu District, Shanghai, China; 2 Shanghai Jiahui International Hospital, 689 Guiping Road, Xuhui District, Shanghai, China; 3 Department of Colorectal Surgery, Xinhua Hospital affiliated with Shanghai Jiaotong University School of Medicine, 1665 Kongjiang Road, Yangpu District, Shanghai, China

**Keywords:** Type 2 diabetes, Colorectal Cancer, Soluble receptor for advanced glycation end-products

## Abstract

**Background:**

Recent study showed that individuals with type 2 diabetes have a high risk of developing colorectal cancer (CRC), in which Receptor for Advanced Glycation End Products (RAGE) plays a pivotal role. We conducted a cross-sectional study to examine the relationships of circulating sRAGE, CRC and other clinical factors in type2 diabetes patients.

**Methods:**

A total of 150 type 2 diabetes patients aged 50 years and older were enrolled, including 50 patients with CRC and 100 patients without CRC. We measured Serum levels of sRAGE and interleukin-6(IL-6) using an enzyme-linked immunosorbent assay (ELISA). In addition, other clinical parameters were also measured during hospitalization.

**Results:**

Type 2 diabetes patients with CRC had higher triglyceride, total cholesterol, IL-6, and circulating sRAGE levels and lower use of medicines than type 2 diabetes patients without CRC. Circulating sRAGE was associated with an increased risk for CRC (OR = 2.289 for each SD increase in sRAGE, 95% CI = 1.037–5.051; *P* = 0.04) among Type 2 diabetes patients after adjustment for confounders. Furthermore, circulating sRAGE levels among type 2 diabetes patients were positively correlated with triglyceride (*r* = 0.377, *P* < 0.001), total cholesterol (*r* = 0.491, *P* < 0.001), and low-density lipoprotein cholesterol (LDL-c)(*r* = 0.330, *P* < 0.001) levels; the homeostatic model assessment for insulin resistance(HOMA-IR)score (*r* = 0.194, *P* = 0.017); and fasting serum insulin (*r* = 0.167, *P* = 0.041) and IL-6 (*r* = 0.311, *P* < 0.001) concentrations.

**Conclusions:**

Our results suggested that circulating sRAGE is independently risk factor for CRC, and also closely related to inflammation, dyslipidemia in type 2 diabetes patients.

## Background

CRC is the third most commonly diagnosed cancer and the second leading cause of cancer death, accounting for 10.2% of the total diagnosed cancer and 9.2% of the cancer death [1]. Multiple risk factors of colorectal cancer, including sedentary lifestyle, obesity and a western-style diet,are closely related to diabetes [2, 3]. Diabetes in adults are becoming increasingly common all over the world, with number of 451 million in 2017 and this number is evaluated to increase to 693 million by 2045 [4]. Several studies have previously reported that individuals with type 2 diabetes have a higher risk of developing CRC than their nondiabetic counterparts [5–8]. Therefore, it’s important to understand the potential pathogenetic links between these two diseases.

The receptor for advanced glycation end products (RAGE) is a multiligand, transmembrane cell surface receptor belonging to the immunoglobulin superfamily [9]; major ligands for RAGE are advanced glycation end products (AGEs), high-mobility group box 1 (HMGB1), S100/calgranulins and amyloid-beta [10]. Binding of RAGE to its ligands can activate chronic inflammatory conditions and create a microenvironment that strongly contributes to tumor development [11, 12]. In our recent study, we demonstrated that AGE–RAGE signaling enhanced HCT116 CRC cell proliferation, in which AGE–RAGE-mediated carbohydrate responsive element binding protein (ChREBP) induction played an important role [13]. In addition to the membrane-bound isoform of RAGE, several soluble forms of RAGE (sRAGE) appear in circulating, generated by endogenous secretory major splice variant of RAGE (esRAGE) or proteolytic cleavage of the cell-bound receptor (cRAGE) [14, 15]. sRAGE binding to RAGE ligands cannot trigger a signaling cascade because it has no transmembrane and intracellular domains. For this reason, sRAGE is thought to play a beneficial role by acting as an invalid receptor that attenuates RAGE-ligand interactions at the cell surface [16]. However, the circulating sRAGE concentrations are too low to capture and eliminate AGEs in diabetes patients [17]. Accumulating evidence has shown that increased levels of circulating sRAGE can as one potential marker for the expression of RAGE and activation of the RAGE axis, resulting many adverse outcomes in diabetes patients [17–19]. Therefore, we hypothesized that type 2 diabetes patients with higher circulating sRAGE are at a higher risk of developing CRC.

In this cross-sectional study, we focused on exploring the relationships between circulating sRAGE levels and CRC and other clinical factors in type 2 diabetes patients, with the aim to find out potential therapeutic interventions for reducing occurrence and progression of CRC in type2 diabetes patients.

## Methods

### Subjects

The participants were hospitalized in the Endocrinology or Colorectal Surgery inpatient department of Xinhua Hospital Affiliated with Shanghai Jiaotong University School of Medicine between October 2016 and January 2018. Informed consent was obtained from all participants. All enrolled subjects had type 2 diabetes aged 50 years and older and type 2 diabetes was diagnosed according to the 1999 World Health Organization criteria. Patients with CRC were diagnosed by histopathology for the first time and without any treatment for CRC. Patients were excluded based on the following criteria: a history of cancer, inflammatory bowel disease, severe cardiovascular disease, chronic renal insufficiency, severe liver dysfunction, a family history of CRC and previous CRC diagnosis. Finally, 150 patients were selected and divided into two groups: 50 diabetes patients (33 men and 17 women) with histologically proven CRC for the first time were divided into the type 2 diabetes patients with CRC group, and the other 100 diabetes patients (54 men and 46 women) without CRC were divided into the type 2 diabetes patients without CRC group. The study was approved by the Ethics Committee of Xinhua Hospital Affiliated with Shanghai Jiaotong University School of Medicine.

### Measurement of circulating sRAGE, IL-6, and other clinical indicators

All serum samples of eligible study subjects were obtained in a fasted state. Serum samples for sRAGE and IL-6 analysis were stored at − 80 °C until analysis. Circulating sRAGE and IL-6 were detected with enzyme-linked immunosorbent assay (ELISA) kits (Human sRAGE Quantikine and Human IL-6 Valukine; R&D Systems, Minneapolis, MN) in duplicate according to the manufacturer’s recommendations. For the ELISAs, the inter-assay coefficient of variation (CV) was 4–10%, and the intra-assay CV was 3–9%.Blood insulin and C-peptide concentrations were assayed by an automated analyzer (ADVIA Centaur XP, Siemens, Berlin, Germany). Hemoglobin A1c (HbA1c) was determined by high-performance liquid chromatography (BIO-RAD VARIANT II, California, USA). Blood glucose and lipid levels were measured with an autoanalyzer (Hitachi 7600, Tokyo, Japan). Height and body weight were measured, body mass index (BMI kg/m^2^) and homeostatic model assessment for insulin resistance (HOMA-IR) were calculated. The current and past medical histories, personal backgrounds and chronic diabetic complications of all participants were investigated by trained physicians during the hospitalization.

### Statistical analyses

The results are expressed as the means ± SDs for normally distributed variables and as medians (interquartile ranges) for nonnormally distributed variables. Categorical variables were expressed as percentages. Data with normal distributions and homoscedasticity were analyzed by Student’s t test, and those with non-normal distributions or heteroscedasticity were analyzed by the Wilcoxon rank sum test. The χ^2^ test was used for categorical variables. We examined the correlation between circulating sRAGE levels and the study variables using Pearson correlation analysis for normally distributed variables and Spearman rank correlation analysis for nonnormally distributed variables. To investigate the associations between circulating sRAGE concentrations and CRC, multivariable adjusted logistic regression analyses were performed to assess the OR for CRC. The variable circulating sRAGE was standardized for the logistic regression analysis. Statistical analyses were performed in SPSS version 22 (IBM Corp, Armonk, NY, USA). A two-sided *p* value of < 0.05 was considered to indicate significance.

## Results

### Characteristics of enrolled patients

Table 1 shows the demographic and clinical characteristics of enrolled type 2 diabetes patients with and without CRC. There were no significant differences between the groups in terms of sex, age, duration of diabetes, BMI, LDL-c level, HDL-c level, HOMA-IR level, fasting plasma glucose level, fasting C-peptide level, fasting serum insulin level, hemoglobin A1c percentage, percentage of current smokers and use of sulfonylurea (all *P* > 0.05). Compared with control participants, type 2 diabetes patients with CRC had significantly higher triglyceride, total cholesterol, IL-6, and circulating sRAGE levels, and a lower proportion of these patients used medicines, including insulin, metformin, thiazolidinedione, a-glucosidase inhibitors, NSAIDs and statins (all *P* < 0.05).

**Table 1:** Characteristics of type 2 diabetes patients with and without CRC

	T2D with CRC	T2D without CRC	*p*
Number of patients	50	100	–
Sex (male %)	66	54	0.160
Age (yr)	69.44 ± 7.73	67.29 ± 7.83	0.114
Duration of diabetes (yr)	11.50 (5.00–15.00)	10.00 (6.00–18.00)	0.743
BMI (kg/m2)	24.70 ± 2.04	25.25 ± 3.21	0.205
TG (mmol/L)	1.98 (1.64–2.33)	1.65 (1.08–2.30)	0.028
TC (mmol/L)	4.78 (4.33–5.04)	4.33 (3.43–4.98)	0.013
LDL-c (mmol/L)	2.64 ± 0.58	2.42 ± 0.83	0.061
HDL-c (mmol/L)	1.16 ± 0.40	1.16 ± 0.27	0.957
HOMA-IR	3.93 (2.43–5.46)	3.66 (1.75–5.61)	0.539
Fasting plasma glucose (mmol/L)	7.41 (5.92–9.47)	7.73 (6.27–9.37)	0.534
Fasting C-peptide (nmol/L)	0.83 (0.66–1.06)	0.74 (0.51–1.05)	0.096
Fasting serum insulin (pmol/L)	83.48 (63.47–109.38)	72.43 (43.22–100.78)	0.191
Hemoglobin A1c (%)	8.09 ± 0.79	8.47 ± 1.77	0.071
IL-6 (ng/L)	78.70 (58.23–114.17)	24.49 (14.00–36.05)	<0.001
sRAGE (ng/L)	604.61 ± 210.21	404.80 ± 166.18	<0.001
Current smoker (%)	26	15	0.103
Insulin use (%)	34	84	<0.001
Sulfonylurea use (%)	32	45	0.127
Metformin use (%)	16	83	<0.001
Thiazolidinedione use (%)	0	13	0.018
a-Glucosidase inhibitor use (%)	12	62	<0.001
NSAID use (%)	8	41	<0.001
Statin use (%)	6	40	<0.001

Student’s t test, the Wilcoxon rank sum test, or the χ^2^ test was used to test for significant differences. *BMI* body mass index, *TG* triglycerides, *TC* total cholesterol, *LDL-c* low-density lipoprotein cholesterol, *HDL-c* high-density lipoprotein cholesterol, *HOMA-IR* homeostatic model assessment for insulin resistance, *IL-6* interleukin-6, *sRAGE* soluble receptor for advanced glycation end products

### The relationship between sRAGE levels and other clinical factors

The circulating sRAGE level was significantly and positively correlated with triglyceride, total cholesterol, and LDL-c levels; the HOMA-IR score; and fasting serum insulin and IL-6 levels (*r* = 0.377, *r* = 0.491, *r* = 0.330, *r* = 0.194, *r* = 0.167, and *r* = 0.311, respectively; all *P* < 0.05). The circulating sRAGE level was not significantly correlated with age, the duration of diabetes, BMI, or the levels of HDL-c, fasting plasma glucose, fasting C-peptide or hemoglobin A1c (all *P* > 0.05) (Table 2).

**Table 2:** Correlations of sRAGE and other clinical parameters in the study subjects

	r	***p***
Age (yr)	0.072	0.380
Duration of diabetes (yr)	−0.144	0.079
BMI (kg/m2)	0.001	0.992
TG (mmol/L)	0.377	<0.001
TC (mmol/L)	0.491	<0.001
LDL-c (mmol/L)	0.330	<0.001
HDL-c (mmol/L)	0.048	0.558
HOMA-IR	0.194	0.017
Fasting plasma glucose (mmol/L)	0.142	0.083
Fasting C-peptide (nmol/L)	0.110	0.181
Fasting serum insulin (pmol/L)	0.167	0.041
Hemoglobin A1c (%)	0.053	0.519
IL-6 (ng/L)	0.311	<0.001

### Influences of medicine use on circulating sRAGE concentration

**Fig. 1:**
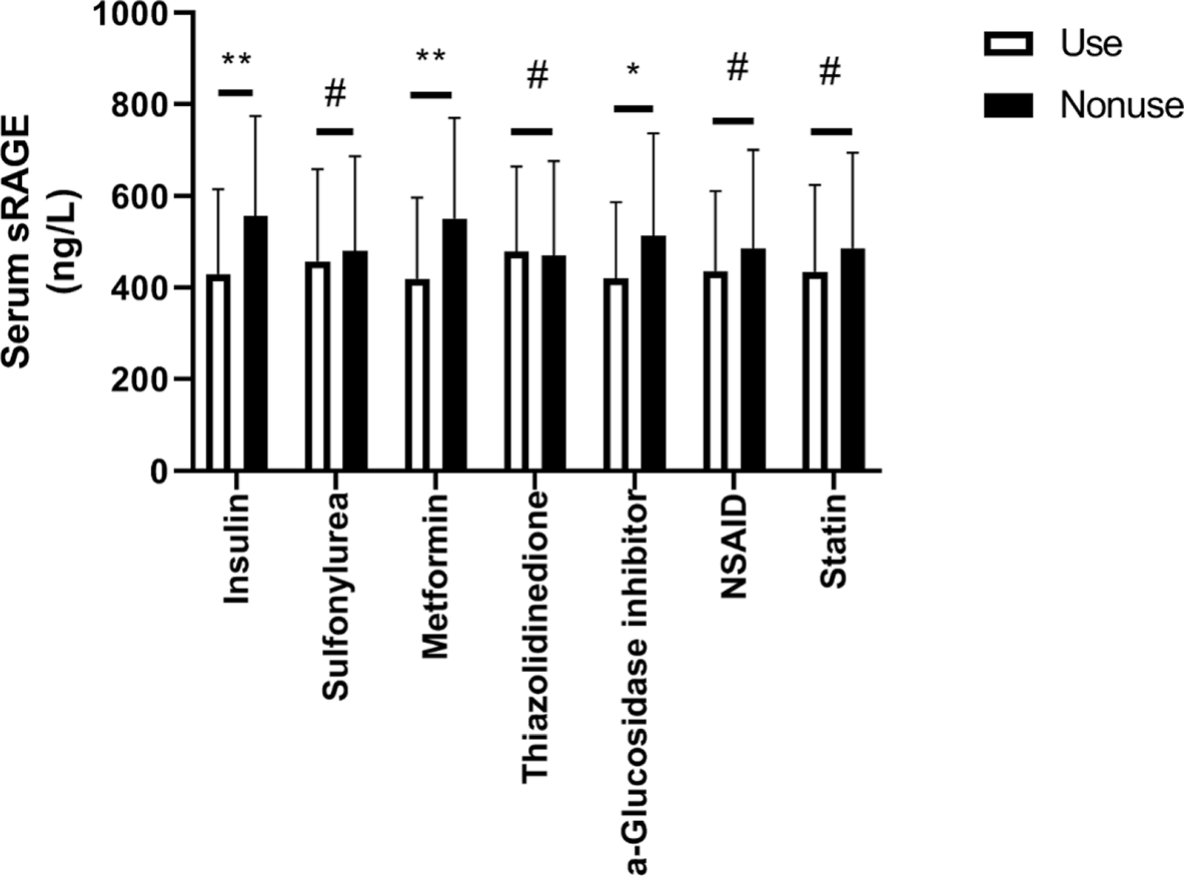
Circulating sRAGE concentration in groups using or not using different drugs. *p<0.05, **p<0.001, #*p*>0.05

Figure 1 shows the influence of different medicines on the circulating sRAGE concentration. Type 2 diabetes patients treated with insulin showed lower circulating sRAGE levels than type 2 diabetes patients not treated with insulin (429.87 ± 185.19 versus 557.03 ± 217.32 ng/L, *P* < 0.001). Type 2 diabetes patients treated with metformin showed lower circulating sRAGE levels than type 2 diabetes patients not treated with metformin (419.87 ± 177.00 versus 550.90 ± 219.55 ng/L, *P* < 0.001), and type 2 diabetes patients treated with a-glucosidase inhibitors showed lower circulating sRAGE levels than type 2 diabetes patients not treated with a-glucosidase inhibitor (420.11 ± 167.05 versus 513.94 ± 223.13 ng/L, *p* = 0.005). The use of sulfonylurea, thiazolidinedione, NSAIDs and statins did not significantly influence circulating sRAGE levels (*P* > 0.05 for all).

### The risk of CRC associated with increased circulating sRAGE levels

Table 3 shows that for subjects with a 1-SD increase in the circulating sRAGE level, the OR for CRC was increased (OR = 2.289; 95% CI = 1.037–5.051; *P* = 0.04) after adjustment for age; sex, BMI, smoking status, HOMA-IR score, and the levels of triglycerides, total cholesterol, LDL-c, HDL-c, and IL-6.

**Table 3:** Risk of CRC associated with a 1-SD increase in the circulating sRAGE level

	OR (95% CI)	*P*
Model 1	3.331 (2.075–5.347)	<0.001
Model 2	3.603 (2.089–6.217)	<0.001
Model 3	3.609 (2.081–6.257)	<0.001
Model 4	2.289 (1.037–5.051)	0.040

*OR* odds ratio, *CI* confidence interval. We assigned the value of 0 to participants without CRC and assigned the value of 1 to those with CRC. Model 1 was adjusted for age, sex, BMI and smoking status. Model 2 was further adjusted for triglyceride, total cholesterol, LDL-c, and HDL-c levels based on model 1. Model 3 was further adjusted for the HOMA-IR score based on model 2. Model 4 was further adjusted for IL-6 levels based on model 3.

## Discussion

Our study of type 2 diabetes patients indicated that higher circulating sRAGE levels were significantly associated with an increased risk of CRC. Moreover, increased circulating sRAGE levels were significantly correlated with increased triglyceride, total cholesterol, LDL-c, fasting serum insulin, and IL-6 levels and HOMA-IR scores. Type 2 diabetes patients treated with insulin, metformin and a-glucosidase inhibitors showed lower circulating sRAGE levels than Type 2 diabetes patients not treated with these agents.

RAGE was recently reported to be highly expressed in CRC tissues and to be closely associated with invasion, metastasis, and angiogenesis in CRC [20, 21]. In this study, we found that the circulating sRAGE level was notably increased in type 2 diabetes patients with CRC. Regression analysis further indicated that an elevated level of circulating sRAGE was independently associated with an increased incidence of CRC in type 2 diabetes patients. Higher level of circulating sRAGR contribute to higher risk of developing CRC mainly related to activation of RAGE axis. RAGE interacts with diverse ligands and activates multiple signaling pathways, which are the main contributors to the development of malignancies. The major RAGE axis-related and underlying molecular mechanisms inducing CRC in patients with type 2 diabetes may include the following: 1) AGE–RAGE interaction promotes the activation of multiple signaling cascades, including the NADPH oxidase, Jak/Stat, and MAPK cascades, resulting in the activation of transcription factors, such as nuclear factor kB (NF-kB), or IFN-stimulated response elements (ISRE), which are involved in proliferation, metastasis, tumor generation [22]. 2) The S100P-RAGE interaction stimulates signaling pathways such as the MAPK, NF-kB, and PI3K/Akt pathways, which are closely related to proliferation and angiogenesis [23, 24]. 3) The HMGB1-RAGE interaction activates signaling pathways, including the K-Ras, MAPK and NF-Κb pathways, which are closely related to inflammation and cancer [25–29]. In addition, RAGE can directly stimulate some proximal signaling events related to cancer [30]. Thus, RAGE plays a vital role in the development of CRC in type 2 diabetes patients through a complex process.

Our results indicate that type 2 diabetes patients with CRC had significantly higher IL-6 levels than controls, revealing associations between IL-6 levels and CRC. Consistent with this result, accumulating evidence indicates that higher IL-6 concentrations are associated with a higher risk for CRC development [31, 32]. As a proinflammatory cytokine, IL-6, along with IL-6 signaling, is closely related to the initiation and progression of the CRC-associated inflammatory microenvironment [33, 34]. Previous studies reported that IL-6 mediates the promotion of tumorigenesis by mainly activating the JAK/STAT3 signaling pathway [35–37]. We also found that circulating sRAGE was positively correlated with IL-6 and that a 1-SD increase in the circulating sRAGE level was associated with a 2.29-fold increase in CRC risk, regardless of the IL-6 level. Consistent with this result, a previous study reported that RAGE signaling pathways can upregulate the expression of important cytokines, including IL-6; and that these cytokines and their respective receptors trigger multiple signaling pathways, resulting in the production of large amounts of proinflammatory mediators and RAGE in a positive feed-forward loop [12]. Both RAGE and IL-6 play vital roles in linking cancer with inflammation through multiple signaling pathways [12]. Thus, measurement of circulating sRAGE and IL-6 level can aid the prediction and/or diagnosis of CRC in middle-aged and older type 2 diabetes patients.

Our results indicate that type 2 diabetes patients with CRC had significantly higher triglyceride and total cholesterol levels than controls, consistent with previous studies reporting that total cholesterol and triglycerides increase the risk of CRC [38, 39]. Although the mechanism underlying the links between total cholesterol and triglycerides and CRC has not been completely clarified, several potential mechanisms have been proposed. The association between triglycerides and CRC may be ascribed to bile acid excretion or the energy supply to neoplastic cells [40]. Furthermore, as the major components of dyslipidemia, total cholesterol and triglycerides are linked to chronic inflammation [41], oxidative stress [42], and insulin resistance [43], all of which are associated with neoplastic processes. In addition, dyslipidemia and CRC have common environmental risk factors, such as Western eating patterns, alcohol use, obesity, and low physical activity levels [40]. In our study, we found that the levels of both cholesterol and triglycerides were positively correlated with the circulating sRAGE level, suggesting that the roles of cholesterol and triglycerides in inducing the development of CRC may be mediated through the RAGE signaling pathway. Further studies are required to better understand the role of dyslipidemia in CRC.

Various antidiabetes agents have different influences on colorectal carcinogenesis. Among these medicines, metformin, a-glucosidase inhibitors and insulin have received the most attention all the time. In patients with diabetes, treatment with metformin and a-glucosidase inhibitors is associated with a reduced risk of developing CRC [44, 45], whereas insulin has been shown to promote CRC [46]. In our study, we found that type 2 diabetes patients treated with insulin, metformin or a-glucosidase inhibitors had lower circulating sRAGE levels than patients not treated with these agents and that type 2 diabetes patients with CRC had lower rates of medicine use, including insulin, metformin, a-glucosidase inhibitors, thiazolidinedione, NSAIDs and statins, suggesting that insulin, metformin and a-glucosidase inhibitors may protect against CRC mediated by RAGE. The potential reason that the results of our study differ from those of previous research is that the hypoglycemic effect of insulin plays a greater role than its proliferative effect in these type 2 diabetes patients with CRC, whose glucose was poorly managed, as reflected by the lower use of most kinds of diabetes medicines. Our results suggest that diabetes management is very important for Type 2 diabetes patients to reduce chronic complications, including CRC.

To our knowledge, this is the first study specifically aimed at exploring the relationships among circulating sRAGE concentrations, CRC and clinical factors in type 2 diabetes patients. However, several limitations should be addressed. First, a cause-effect relationship cannot be inferred because of the cross-sectional nature of this study. Second, information about the cancer stages, tumor sites and histopathological outcomes of the patients with CRC was insufficient. Including these assessments may improve the strength of future studies. Third, since the subjects were recruited from a single center in China, the study population may not represent the general population.

## Conclusions

In conclusion, our results suggested that circulating sRAGE is independently risk factor for CRC, and also closely related to inflammation, dyslipidemia in type 2 diabetes patients. Therapeutic interventions that reduce RAGE levels or RAGE axis activity deserve further investigation.

## Data Availability

The data that support the findings of this study are available from the corresponding author on reasonable request. Inquiries for data access may be sent to the following e-mail address: chenhanbei@xinhuamed.com.cn

## Abbreviations

CRC: colorectal cancer
sRAGE: Soluble Receptor for Advanced Glycation End Products
ChREBP: Carbohydrate responsive element binding protein
BMI: Body mass index
TG: Triglycerides
TC: Total cholesterol
LDL-c: Low-density lipoprotein cholesterol
HDL-c: High-density lipoprotein cholesterol
HOMA-IR: Homeostatic model assessment for insulin resistance
IL-6: Interleukin-6

## References

[1] Bray F, Ferlay J, Soerjomataram I, Siegel RL, Torre LA, Jemal A (2018). Global cancer statistics 2018: GLOBOCAN estimates of incidence and mortality worldwide for 36 cancers in 185 countries. CA Cancer J Clin.

[2] Saetang J, Sangkhathat S (2017). Diets link metabolic syndrome and colorectal cancer development (review). Oncol Rep.

[3] Yuhara H, Steinmaus C, Cohen SE, Corley DA, Tei Y, Buffler PA. Is diabetes mellitus an independent risk factor for colon cancer and rectal cancer? Am J Gastroenterol 2011;106(11):1911–1921; quiz 22. 10.1038/ajg.2011.301 PMC3741453 21912438

[4] Cho NH, Shaw JE, Karuranga S, Huang Y, da Rocha Fernandes JD, Ohlrogge AW (2018). IDF diabetes atlas: global estimates of diabetes prevalence for 2017 and projections for 2045. Diabetes Res Clin Pract.

[5] Peeters PJHL, Bazelier MT, Leufkens HGM, de Vries F, De Bruin ML (2015). The risk of colorectal cancer in patients with type 2 diabetes: associations with treatment stage and obesity. Diabetes Care.

[6] Larsson SC, Giovannucci E, Wolk A (2005). Diabetes and colorectal cancer incidence in the cohort of Swedish men. Diabetes Care.

[7] He J, Stram DO, Kolonel LN, Henderson BE, Le Marchand L, Haiman CA (2010). The association of diabetes with colorectal cancer risk: the multiethnic cohort. Br J Cancer.

[8] Limburg PJ, Vierkant RA, Fredericksen ZS, Leibson CL, Rizza RA, Gupta AK (2006). Clinically confirmed type 2 diabetes mellitus and colorectal cancer risk: a population-based, retrospective cohort study. Am J Gastroenterol.

[9] Neeper M, Schmidt AM, Brett J, Yan SD, Wang F, Pan YC (1992). Cloning and expression of a cell surface receptor for advanced glycosylation end products of proteins. J Biol Chem.

[10] Xie J, Mendez JD, Mendez-Valenzuela V, Aguilar-Hernandez MM (2013). Cellular signalling of the receptor for advanced glycation end products (RAGE). Cell Signal.

[11] Ahmad S, Khan H, Siddiqui Z, Khan MY, Rehman S, Shahab U (2018). AGEs, RAGEs and s-RAGE; friend or foe for cancer. Semin Cancer Biol.

[12] Riehl A, Németh J, Angel P, Hess J (2009). The receptor RAGE: bridging inflammation and cancer. Cell Commun Signal.

[13] Chen H, Wu L, Li Y, Meng J, Lin N, Yang D (2014). Advanced glycation end products increase carbohydrate responsive element binding protein expression and promote cancer cell proliferation. Mol Cell Endocrinol.

[14] Yonekura H, Yamamoto Y, Sakurai S, Petrova RG, Abedin MJ, Li H (2003). Novel splice variants of the receptor for advanced glycation end-products expressed in human vascular endothelial cells and pericytes, and their putative roles in diabetes-induced vascular injury. Biochem J.

[15] Raucci A, Cugusi S, Antonelli A, Barabino SM, Monti L, Bierhaus A (2008). A soluble form of the receptor for advanced glycation endproducts (RAGE) is produced by proteolytic cleavage of the membrane-bound form by the sheddase a disintegrin and metalloprotease 10 (ADAM10). FASEB J.

[16] Vazzana N, Santilli F, Cuccurullo C, Davì G (2009). Soluble forms of RAGE in internal medicine. Intern Emerg Med.

[17] Nakamura K, Yamagishi S, Adachi H, Matsui T, Kurita-Nakamura Y, Takeuchi M (2008). Serum levels of soluble form of receptor for advanced glycation end products (sRAGE) are positively associated with circulating AGEs and soluble form of VCAM-1 in patients with type 2 diabetes. Microvasc Res.

[18] Thomas MC, Woodward M, Neal B, Li Q, Pickering R, Marre M (2015). Relationship between levels of advanced glycation end products and their soluble receptor and adverse outcomes in adults with type 2 diabetes. Diabetes Care.

[19] Colhoun HM, Betteridge DJ, Durrington P, Hitman G, Neil A, Livingstone S (2011). Total soluble and endogenous secretory receptor for advanced glycation end products as predictive biomarkers of coronary heart disease risk in patients with type 2 diabetes: an analysis from the CARDS trial. Diabetes..

[20] Liang H, Zhong Y, Zhou S, Peng L (2011). Knockdown of RAGE expression inhibits colorectal cancer cell invasion and suppresses angiogenesis in vitro and in vivo. Cancer Lett.

[21] Kuniyasu H, Chihara Y, Takahashi T (2003). Co-expression of receptor for advanced glycation end products and the ligand amphoterin associates closely with metastasis of colorectal cancer. Oncol Rep.

[22] Ott C, Jacobs K, Haucke E, Navarrete Santos A, Grune T, Simm A (2014). Role of advanced glycation end products in cellular signaling. Redox Biol.

[23] Fuentes MK, Nigavekar SS, Arumugam T, Logsdon CD, Schmidt AM, Park JC (2007). RAGE activation by S100P in colon cancer stimulates growth, migration, and cell signaling pathways. Dis Colon Rectum.

[24] Seguella L, Capuano R, Pesce M, Annunziata G, Pesce M, de Conno B, et al. S100B Protein Stimulates Proliferation and Angiogenic Mediators Release through RAGE/pAkt/mTOR Pathway in Human Colon Adenocarcinoma Caco-2 Cells. Int J Mol Sci. 2019;20(13):3240. 10.3390/ijms20133240 PMC6651655 31266264

[25] Qian F, Xiao J, Gai L, Zhu J (2019). HMGB1-RAGE signaling facilitates Ras-dependent Yap1 expression to drive colorectal cancer stemness and development. Mol Carcinog.

[26] Huang CY, Chiang SF, Chen WT, Ke TW, Chen TW, You YS (2018). HMGB1 promotes ERK-mediated mitochondrial Drp1 phosphorylation for chemoresistance through RAGE in colorectal cancer. Cell Death Dis.

[27] Huang M, Geng Y, Deng Q, Li R, Shao X, Zhang Z (2018). Translationally controlled tumor protein affects colorectal cancer metastasis through the high mobility group box 1-dependent pathway. Int J Oncol.

[28] Zhu L, Li X, Chen Y, Fang J, Ge Z (2015). High-mobility group box 1: a novel inducer of the epithelial-mesenchymal transition in colorectal carcinoma. Cancer Lett.

[29] Sims GP, Rowe DC, Rietdijk ST, Herbst R, Coyle AJ (2010). HMGB1 and RAGE in inflammation and cancer. Annu Rev Immunol.

[30] Ishihara K, Tsutsumi K, Kawane S, Nakajima M, Kasaoka T (2003). The receptor for advanced glycation end-products (RAGE) directly binds to ERK by a D-domain-like docking site. FEBS Lett.

[31] Kim J, Lee J, Oh JH, Chang HJ, Sohn DK, Shin A, et al. Circulating Interleukin-6 Level, Dietary Antioxidant Capacity, and Risk of Colorectal Cancer. Antioxidants (Basel). 2019;8(12):595. 10.3390/antiox8120595 PMC6943549 31795177

[32] Chung YC, Chang YF (2003). Serum interleukin-6 levels reflect the disease status of colorectal cancer. J Surg Oncol.

[33] Hunter CA, Jones SA (2015). IL-6 as a keystone cytokine in health and disease. Nat Immunol.

[34] Mauer J, Denson JL, Bruning JC (2015). Versatile functions for IL-6 in metabolism and cancer. Trends Immunol.

[35] Waldner MJ, Foersch S, Neurath MF (2012). Interleukin-6--a key regulator of colorectal cancer development. Int J Biol Sci.

[36] Grivennikov S, Karin E, Terzic J, Mucida D, Yu GY, Vallabhapurapu S (2009). IL-6 and Stat3 are required for survival of intestinal epithelial cells and development of colitis-associated cancer. Cancer Cell.

[37] De Simone V, Franze E, Ronchetti G, Colantoni A, Fantini MC, Di Fusco D (2015). Th17-type cytokines, IL-6 and TNF-alpha synergistically activate STAT3 and NF-kB to promote colorectal cancer cell growth. Oncogene..

[38] Rodriguez-Broadbent H, Law PJ, Sud A, Palin K, Tuupanen S, Gylfe A (2017). Mendelian randomisation implicates hyperlipidaemia as a risk factor for colorectal cancer. Int J Cancer.

[39] Yao X, Tian Z (2015). Dyslipidemia and colorectal cancer risk: a meta-analysis of prospective studies. Cancer Causes Control.

[40] McKeown-Eyssen G (1994). Epidemiology of colorectal cancer revisited: are serum triglycerides and/or plasma glucose associated with risk?. Cancer Epidemiol Biomark Prev.

[41] Esteve E, Ricart W, Fernandez-Real JM (2005). Dyslipidemia and inflammation: an evolutionary conserved mechanism. Clin Nutr.

[42] Kontush A, de Faria EC, Chantepie S, Chapman MJ (2005). A normotriglyceridemic, low HDL-cholesterol phenotype is characterised by elevated oxidative stress and HDL particles with attenuated antioxidative activity. Atherosclerosis..

[43] Avramoglu RK, Basciano H, Adeli K (2006). Lipid and lipoprotein dysregulation in insulin resistant states. Clin Chim Acta.

[44] Sehdev A, Shih YC, Vekhter B, Bissonnette MB, Olopade OI, Polite BN (2015). Metformin for primary colorectal cancer prevention in patients with diabetes: a case-control study in a US population. Cancer..

[45] Tseng Y-H, Tsan Y-T, Chan W-C, Sheu WH-H, Chen P-C (2015). Use of an α-Glucosidase inhibitor and the risk of colorectal Cancer in patients with diabetes: a Nationwide, Population-Based Cohort Study. Diab Care.

[46] Yang YX, Hennessy S, Lewis JD (2004). Insulin therapy and colorectal cancer risk among type 2 diabetes mellitus patients. Gastroenterology..

